# Enhanced Subtyping Scheme for *Salmonella* Enteritidis

**DOI:** 10.3201/eid1312.070185

**Published:** 2007-12

**Authors:** Jie Zheng, Christine E. Keys, Shaohua Zhao, Jianghong Meng, Eric W. Brown

**Affiliations:** * University of Maryland, College Park, Maryland, USA; † US Food and Drug Administration, College Park, Maryland, USA; ‡ US Food and Drug Administration, Laurel, Maryland, USA

**Keywords:** Salmonella Enteritidis, subtyping, differentiation, pulsed-field gel electrophoresis, molecular epidemiology, clone, restriction endonuclease, genetic diversity, dispatch

## Abstract

To improve pulsed-field gel electrophoresis–based strain discrimination of 76 *Salmonella* Enteritidis strains, we evaluated 6 macro-restriction endonucleases, separately and in various combinations. One 3-enzyme subset, *Sfi*I/*Pac*I/*Not*I, was highly discriminatory. Five different indices, including the Simpson diversity index, supported this 3-enzyme combination for improved differentiation of *S*. Enteritidis.

Since 1987, *Salmonella* Enteritidis has been one of the most frequently isolated salmonellae associated with foodborne outbreaks (*1*). Illness from *S.* Enteritidis is linked to consumption of chickens, eggs, and foods that contain eggs (*2*). *S.* Enteritidis presents an interesting challenge from an epidemiologic perspective. Several reports documented a limited number of genotypes among ecologically diverse *S.* Enteritidis, reinforcing the notion that most *S.* Enteritidis strains are derived from a few endemic clones (*3*,*4*).

Pulsed-field gel electrophoresis (PFGE) is an integral subtyping tool used by several national public health networks (e.g., PulseNet, FoodNet, and VetNet) to differentiate outbreak strain clusters (*5*). The genetic homogeneity of *S.* Enteritidis, however, confounds many subtyping approaches, including PFGE (*6*,*7*). Conventional PFGE protocols lack discriminatory power to cull the subtle genotypic differences that distinguish *S.* Enteritidis strains. A more discriminatory scheme that incorporates combinations of potentially more informative enzymes may be attainable. We explored the discriminatory power of 6 enzymes, individually and in combination, to identify a more informative PFGE-based subtyping scheme for this important foodborne pathogen.

## The Study

We examined 76 strains of *S.* Enteritidis and 74 strains of *S.* Typhimurium. Strains were isolated from poultry and poultry-related sources and were obtained from the Center for Veterinary Medicine and Center for Food Safety and Applied Nutrition of the US Food and Drug Administration and from the University of Georgia. After screening numerous restriction enzymes, the 6 selected were *Xba*I, *Bln*I, and *Spe*I, all used in PulseNet protocols (*5*); *Sfi*I and *Pac*I, previously used to improve resolution in PFGE studies involving *Escherichia coli* O157:H7 (*8*,*9*); and *Not*I, found to yield an optimal number of cut sites (*10*). The standard PulseNet PFGE protocol for non-typhoidal *Salmonella* was performed as described (*11*,*12*). Individual run conditions are listed in Table 1.

**Table 1:** Pulsed-field gel electrophoresis run conditions for 6 restriction enzymes used to subtype *Salmonella* Enteritidis*

Enzyme	Digestion temperature, °C	Enzyme units, per plug	Run time, h†	Initial switch time, s	Final switch time, s
*Xba*I	37	50	19	2.16	63.8
*Bln*I	37	30	19	2.16	63.8
*Spe*I	37	30	20.5	5	25
*Sfi*I	50	30	20.5	5	25
*Pac*I	37	30	20.5	0.1	15
*Not*I	37	30	20.5	0.1	15

*All digestion times were 2–3 h. †Digestions were separated in 1% agarose gels (Cambrex, Baltimore, MD, USA) at 6V/cm by using a CHEF-Mapper (Bio-Rad, Hercules, CA, USA).

Five diversity indices were used to assess discriminatory potential among enzymes. First, unique PFGE patterns or pattern combinations, when analyzing >2 enzymes, were tallied. Second, the mean number of strains per polytomy (an unresolved strain cluster) was calculated as the number of polytomous strains divided by the number of polytomies in the tree. Third, the percentage of polytomous strains (of 76 *S.* Enteritidis strains) was calculated. Fourth, the node:strain ratio was calculated as the number of nodes (bifurcating tree forks) divided by 76 *S.* Enteritidis strains. A node:strain value closer to 1 indicated a more resolved tree. Finally, the Simpson diversity index was calculated as a numerical assessment of the relative discriminatory potential of each enzyme and enzyme combination (*13*).

**Figure 1:**
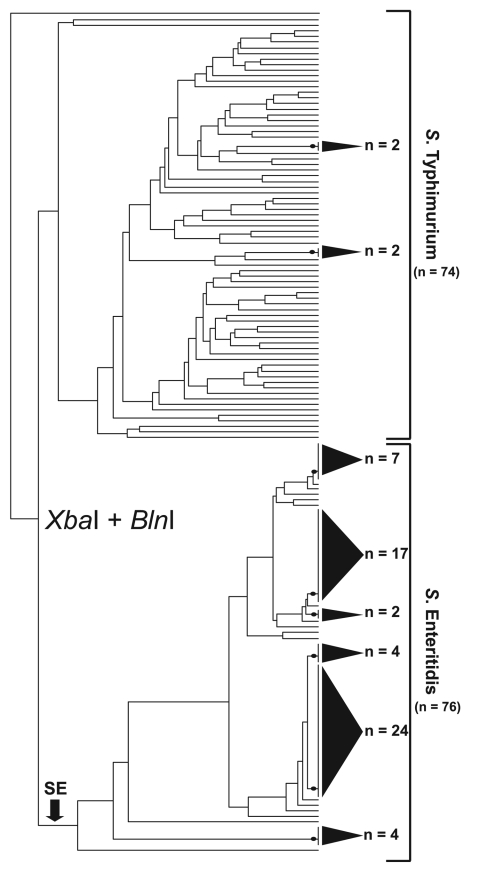
Simultaneous cluster analysis of *Salmonella* Enteritidis and *S*. Typhimurium that used a standard *Xba*I/*Bln*I combined PFGE protocol. The dendrogram incorporates 76 *S.* Enteritidis strains and 74 *S. Typhimurium* strains and depicts the contrasting ability of pulsed-field gel electrophoresis (PFGE) to genetically differentiate these 2 *Salmonella* subspecies I serovars. The dendrogram was generated in BioNumerics v.4.061 (Applied Maths, Sint-Martens-Latem, Belgium) by using band-matched *Xba*I/*Bln*I PFGE data in conjunction with an unweighted pair group method with arithmetic mean clustering algorithm and a Dice similarity coefficient. Shaded cones to the right of terminal tree branches denote polytomies within the dendrogram; adjacent numbers (n) show the strain totals composing that polytomy. An arrow near the bottom of the tree denotes the basal branch of the *S.* Enteritidis cluster. The *S.* Enteritidis portion of the dendrogram comprises strains isolated from Georgia (n = 31), Maryland (n = 8), Pennsylvania (n = 3), Connecticut (n = 3), North Carolina (n = 2), Iowa (n = 2), Tennessee (n = 2), Minnesota (n = 1), Mexico (n = 11), and the People’s Republic of China (n = 6).

*Xba*I-*Bln*I patterns from 76 *S.* Enteritidis strains and 74 *S.* Typhimurium strains were analyzed simultaneously for a direct comparison of PFGE diversity. The resultant dendrogram yielded striking topologic differences between the 2 serovars (Figure 1). *S. Typhimurium* strains were almost entirely resolved; nearly every strain possessed its own branch on the dendrogram. In contrast, *S.* Enteritidis strain discrimination was sharply weaker, affirming extensive genetic homogeneity among strains. For example, 6 polytomies were evident in the *S.* Enteritidis portion of the tree, 5 of which comprised 4 or more strains and 1 of which comprised 24 strains. In total, 76% of *S.* Enteritidis strains occupied unresolved clusters with an average of ≈10 strains per cluster. Moreover, the *S*. Typhimurium dendrogram retained a nearly 1:1 ratio of nodes to strains, indicating that almost every strain retained a unique *Xba*I/*Bln*I pattern combination. *S.* Enteritidis, however, yielded a node:strain ratio of 1:3, indicating a relatively poorly bifurcated tree. Together, these observations highlighted the difficulty in differentiating *S.* Enteritidis with conventional PFGE approaches.

To develop a more discriminatory scheme for *S.* Enteritidis, we examined pattern diversity for 4 additional restriction endonucleases (*Spe*I, *Sfi*I, *Pac*I, and *Not*I). Diversity indices associated with each of the 6 enzymes are listed in Table 2. Many of the indices designated *Not*I as being effective for discriminating *S.* Enteritidis. Among the 6 enzymes, *Not*I yielded the highest number of unique patterns (n = 26), the fewest average number of strains per polytomy (5.2), the lowest percentage of strains captured by polytomies (82%), the highest node:strain ratio (0.47), and the highest Simpson diversity value (0.92). *Not*I was followed closely by *Pac*I for most indices, which suggests that *Pac*I was also useful for *S.* Enteritidis strain discrimination.

**Table 2:** PFGE diversity indices for various combinations of restriction enzymes in *Salmonella* Enteritidis

Enzyme/combination	PFGE patterns*	Mean no. strains/ polytomy†	% polytomous strains‡	Node:strain ratio§	Simpson diversity index
*Xba*I	19	9.1	84	0.30	0.83
*Bln*I	15	16.3	85	0.22	0.76
*Spe*I	16	9.6	88	0.28	0.80
*Sfi*I	15	13.2	87	0.24	0.67
*Pac*I	20	9.0	83	0.32	0.74
*Not*I	26	5.2	82	0.47	0.92
*Xba*I/*Bln*I	24	9.7	76	0.37	0.84
*Xba*I/*Spe*I	26	6.6	78	0.43	0.88
*Xba*I/*Sfi*I	30	5.2	75	0.51	0.92
*Xba*I/*Pac*I	30	5.6	74	0.50	0.91
*Xba*I/*Not*I	38	4.2	66	0.63	0.95
*Bln*I/*Spe*I	22	8.6	79	0.36	0.84
*Bln*I/*Sfi*I	26	6.6	78	0.43	0.89
*Bln*I/*Pac*I	28	6.3	75	0.46	0.88
*Bln*I/*Not*I	36	4.3	68	0.61	0.94
*Spe*I/*Sfi*I	27	5.9	78	0.46	0.90
*Spe*I/*Pac*I	30	6.8	71	0.46	0.89
*Spe*I/*Not*I	35	3.9	72	0.61	0.96
*Sfi*I/*Pac*I	30	5.2	75	0.51	0.87
*Sfi*I/*Not*I	43	4.0	58	0.67	0.96
*Pac*I/*Not*I	42	3.6	62	0.70	0.96
*Spe*I/*Sfi*I/*Not*I	48	3.8	50	0.64	0.97
*Spe*I/*Pac*I/*Not*I	47	3.6	53	0.72	0.97
*Sfi*I/*Pac*I/*Not*I	51	3.3	47	0.79	0.98
6 enzymes	57	3.6	38	0.78	0.98

*PFGE, pulsed-field gel electrophoresis. Absolute tally of unique PFGE fingerprints or fingerprint combinations derived from the corresponding enzyme or group of enzymes. †Total no. polytomous strains/no. polytomies in the dendrogram. ‡No. polytomous strains/76 (total *S.* Enteritidis strains in the analysis). §Total no. nodes in the dendrogram/76 *S.*Enteritidis strains. ¶Derived from the formula for assessing discriminatory capability of a subtyping method (*13*).

Previous studies that used PFGE noted the combining of restriction enzyme data into a single analysis as an approach for improving strain differentiation (*8*,*9*). In our study, a dendrogram of the combined 6-enzyme *S.* Enteritidis data was highly resolved and yielded 57 unique pattern combinations. The tree contained, on average, 3.6 strains per polytomy (n = 8), and only 38% of the strains in the 6-enzyme tree were associated with unresolved clusters (Table 2). The node:strain ratio was 0.78, and the Simpson index was 0.98, surpassing the accepted threshold (0.95) for a useful subtyping scheme (*14*).

Although highly effective for *S.* Enteritidis discrimination, 6-enzyme simultaneous analysis would increase the time and resources needed to complete investigations. Thus, a streamlined PFGE scheme with enhanced discrimination was desirable. First, 2-enzyme combinations were assessed to ascertain a minimal enzyme set for useful discrimination of *S.* Enteritidis. Combinations *Sfi*I/*Not*I and *Pac*I/*Not*I yielded optimal diversity measures, compared with the others (Table 2). However, because no 2-enzyme pair rivaled the discriminatory potential of the 6-enzyme analysis, 3-enzyme combinations were explored. A *Sfi*I/*Pac*I/*Not*I combination was found to be superior in all diversity categories (Table 2). It yielded 51 unique pattern combinations, an average of 3.3 strains per unresolved cluster, a node:strain ratio of 0.79, and a Simpson index of 0.98. The latter 3 measures matched or exceeded corresponding values in the 6-enzyme analysis, which indicates that most of the diversity in the 6-enzyme analysis was captured by this 3-enzyme combination.

**Figure 2:**
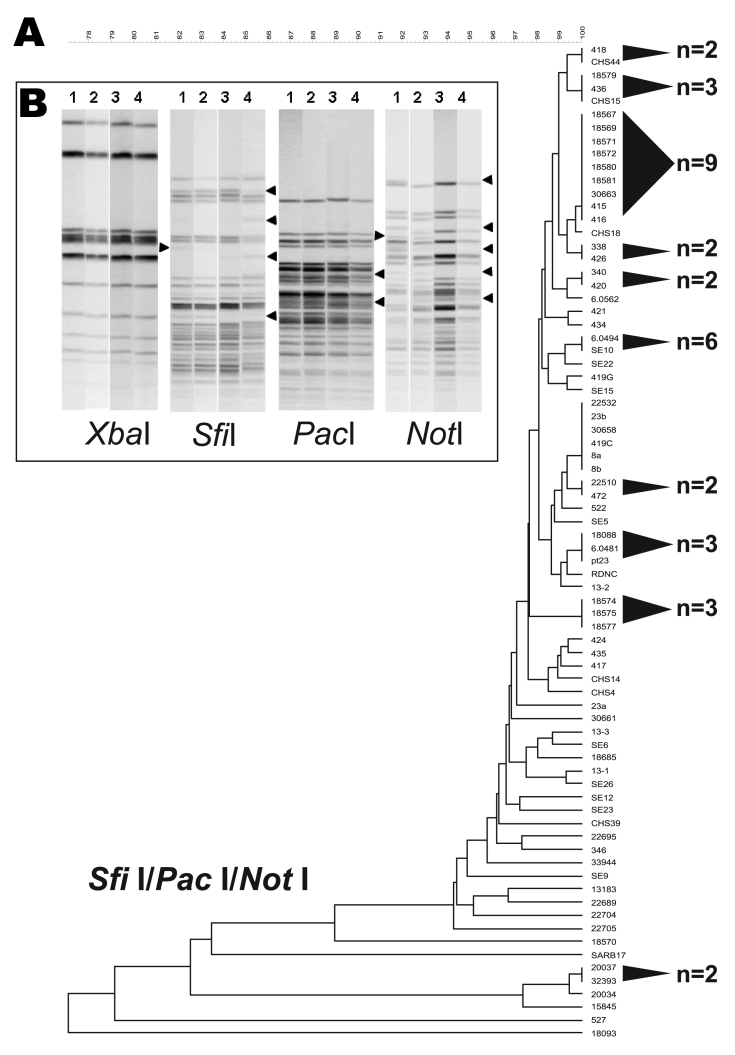
A 3-enzyme pulsed-field gel electrophoresis (PFGE)–based discriminatory scheme of *Salmonella* Enteritidis. A) Dendrogram derived from the combined analysis of PFGE data from *Sfi*I, *Pac*I, and *Not*I. Shaded cones to the right of the terminal branches denote polytomies within each dendrogram; adjacent numbers (n) show the strain totals composing their respective polytomies. A scale depicting percent divergence is presented above the dendrogram. B) Examples of *S.* Enteritidis strain differentiation that used *Sfi*I, *Pac*I, and *Not*I PFGE patterns. The 4 strains are numbered above the gel lanes as follows: 1, *S.* Enteritidis 9; 2, *S.* Enteritidis 12; 3, 22,704; and 4, 22,705. These strains yielded identical PFGE patterns for *Xba*I and *Bln*I. *Xba*I patterns shown here retain no variation among fragments. *Sfi*I, *Pac*I, and *Not*I showed examples of band polymorphism among DNA fragments.

The *Sfi*I/*Pac*I/*Not*I dendrogram showed a highly resolved tree topology and earmarked this 3-enzyme combination as being particularly effective for differentiating *S.* Enteritidis strains (Figure 2, panel A). Of 11 clusters, 9 comprised 3 or fewer strains, and only 47% of *S.* Enteritidis strains composed these 11 clusters, which, aside from the 6-enzyme analysis, represented the lowest portion of polytomous strains in the study.

The robust discrimination of *S.* Enteritidis achieved from combining *Sfi*I, *Pac*I, and *Not*I was attributed to polymorphic band classes that were lacking in other enzymes. As an example, the *Sfi*I, *Pac*I, and *Not*I PFGE patterns for 4 strains (*S.* Enteritidis 9, *S.* Enteritidis 12, 22704, and 22705) that were identical using *Xba*I and *Bln*I were examined for band variation. In contrast to *Xba*I, *Sfi*I, *Pac*I, and *Not*I, all showed some level of polymorphism among DNA fragments (Figure 2, panel B). Band differences for these 3 enzymes partitioned the 4 *S.* Enteritidis strains into disparate dendrogram positions.

## Conclusions

On the basis of common geography and *Xba*I/*Bln*I pattern homogeneity, several clusters of *S.* Enteritidis strains appeared to exhibit clonal relatedness. However, the combined analysis of *Sfi*I/*Pac*I/*Not*I PFGE patterns was able to differentiate not only geographically disparate *S.* Enteritidis strains but also *S.* Enteritidis isolates from within a specific geographic locale (e.g., Georgia *S.* Enteritidis strains 415–421 and 434–436). Although several parameters, such as variable run conditions and times, likely preclude application to outbreak events in real time, the revised scheme presented here may be useful for retrospective epidemiologic investigations in which a more specific focus is often placed on a limited number of epidemiologically related isolates (*15*). Specifically, when *S.* Enteritidis strains are tightly linked geographically and temporally by clonal expansion, this 3-enzyme approach should provide an effective differentiation process.
